# Identification of rice cornichon as a possible cargo receptor for the Golgi-localized sodium transporter OsHKT1;3

**DOI:** 10.1093/jxb/erv069

**Published:** 2015-03-07

**Authors:** Paul Rosas-Santiago, Daniel Lagunas-Gómez, Bronwyn J. Barkla, Rosario Vera-Estrella, Sylvie Lalonde, Alexander Jones, Wolf B. Frommer, Olga Zimmermannova, Hana Sychrová, Omar Pantoja

**Affiliations:** ^1^Instituto de Biotecnología, Universidad Nacional de Autónoma de México, Cuernavaca, Morelos 62250, México; ^2^Department of Plant Biology, Carnegie Institution for Science, Stanford, CA 94305, USA; ^3^Southern Cross Plant Science, Southern Cross University, Lismore, Australia; ^4^Department of Membrane Transport, Institute of Physiology, Academy of Sciences of the Czech Republic, v.v.i., 142 20 Prague 4, Czech Republic

**Keywords:** Cornichon, endoplasmic reticulum, Golgi, OsHKT1;3, protein–protein interaction.

## Abstract

Membrane proteins are synthesized and folded in the endoplasmic reticulum (ER), and continue their path to their site of residence along the secretory pathway. The COPII system has been identified as a key player for selecting and directing the fate of membrane and secretory cargo proteins. Selection of cargo proteins within the COPII vesicles is achieved by cargo receptors. The cornichon cargo receptor belongs to a conserved protein family found in eukaryotes that has been demonstrated to participate in the selection of integral membrane proteins as cargo for their correct targeting. Here it is demonstrated at the cellular level that rice cornichon *Os*CNIH1 interacts with *Os*HKT1;3 and, in yeast cells, enables the expression of the sodium transporter to the Golgi apparatus. Physical and functional HKT–cornichon interactions are confirmed by the mating-based split ubiquitin system, bimolecular fluorescence complementation, and *Xenopus* oocyte and yeast expression systems. The interaction between the two proteins occurs in the ER of plant cells and their co-expression in oocytes leads to the sequestration of the transporter in the ER. In the yeast cornichon mutant *erv14*, *Os*HKT1;3 is mistargeted, preventing the toxic effects of sodium transport in the cell observed in wild-type cells or in the *erv14* mutant that co-expressed *Os*HKT1;3 with either *Os*CNIH1 or Erv14p. Identification and characterization of rice cornichon as a possible cargo receptor opens up the opportunity to improve our knowledge on membrane protein targeting in plant cells.

## Highlight

The cargo receptor cornichon, located in the endoplasmic reticulum, interacts with the low-affinity Na^+^ transporter *Os*HKT1;3 for its delivery to the Golgi apparatus.

## Introduction

Membrane transport proteins have to be selectively targeted to specific membranes to control ion and metabolite fluxes into and within the cell. However, very little is known about the sorting mechanisms of plant transporters. During evolution, eukaryotic cells developed multiple systems to target proteins to their respective compartments. Once membrane proteins are synthesized and folded in the endoplasmic reticulum (ER), they are directed by specific protein–protein interactions through the secretory pathway to the site where they will finally dwell. Transport of membrane and secretory cargo proteins is mediated by COPII vesicles that help to select these cargoes from ER-resident proteins. In yeast, humans, and plants, there is evidence that selection of cargo proteins is mediated specifically by the Sec24 subunit of the COPII complex (Miller *et al.*, 2002; Mossessova *et al.*, 2003; Mancias and Goldberg, 2008; Faso *et al.*, 2009; Conger *et al.*, 2011). Conserved domains involved in cargo selection have been identified (Miller *et al.*, 2003; Mossessova *et al.*, 2003). Accessory proteins known as cargo participate in the selection of specific cargo that can be either soluble or membrane proteins destined for different organelle or vesicular membranes (Dancourt and Barlowe, 2010). Correct targeting of membrane proteins, in particular ion transporters, is important for controlling ion homeostasis within the cell. Participation of cargo receptors is proposed to increase the specificity of the secretory pathway, with a particular receptor recognizing a specific cargo to be delivered to a precise cellular site of residence (Dancourt and Barlowe, 2010). In yeast, the role of Erv proteins (endoplasmic reticulum vesicle proteins) as cargo receptors is indicated by their association with COPI and COPII vesicles, their cycling between the ER and Golgi, and the selective inhibition of cargo transport with the deletion of a particular Erv cargo receptor (Belden and Barlowe, 2001). For example, Erv29p has been demonstrated to select soluble secretory proteins such as carboxypeptidase Y and proteinase A (Belden and Barlowe, 2001; Otte and Barlowe, 2004), while Erv26p is involved in the export of membrane proteins such as alkaline phosphatase and mannosyltransferase (Bue *et al.*, 2006; Bue and Barlowe, 2009). In contrast, Erv14p participates in the ER export of membrane proteins through ER lumen-localized sorting signals (Powers and Barlowe, 1998, 2002). Yeast Erv14p is a well conserved protein found in eukaryotes, initially identified in *Drosophila* as cornichon (Roth *et al.*, 1995), and later in mammals where it was found to be associated with glutamate receptors of the AMPA subtype (Schwenk *et al.*, 2009). In plants, there is little information on the possible role of supplementary cargo receptors in the secretory pathway, with the exception of vacuolar cargo receptor proteins directly involved in the selection of soluble proteins for their delivery to the vacuole lumen (Sanderfoot *et al.*, 1998; Tse *et al.*, 2004; Wang *et al.*, 2011). However, it has been demonstrated in plant transporters that the presence of diacidic (Mikosch *et al.*, 2006; Dunkel *et al.*, 2008) or dileucine motifs (Komarova *et al.*, 2012; Wolfenstetter *et al.*, 2012) or loops between transmembrane domains (Komarova *et al.*, 2012) is important for the correct delivery to the target membrane.

While the acronym HKT for high-affinity K^+^ transporter implies that members of the family transport K^+^, the majority of these proteins play a more significant role in low-affinity Na^+^ transport and have been identified as key players in salt tolerance (Plett *et al.*, 2010; Munns *et al.*, 2012; Schroeder *et al.*, 2013). Yet, knowledge of the regulation of these transporters is limited. HKT transporters form two distinct subfamilies. Members of subfamily 1 are associated with Na^+^ tolerance based on their expression in the parenchyma cells of the xylem where they remove Na^+^ from the root xylem (Uozumi *et al.*, 2000; Ren *et al.*, 2005; Sunarpi *et al.*, 2005) or the leaf sheath (Cotsaftis *et al.*, 2012; Munns *et al.*, 2012), preventing its transport to the leaf blade. Alternatively, they may participate in Na^+^ recirculation from the leaves to the root along the phloem (Berthomieu *et al.*, 2003). The specific overexpression of *At*HKT1;1 in the root stele of *Arabidopsis thaliana* (Møller *et al.*, 2009) or rice (*Oryza sativa*) (Plett *et al.*, 2010) caused a decreased Na^+^ transport to the shoot, with a consequent lower accumulation of Na^+^ in the leaves and higher fresh weight, and resulting in an increase in salinity tolerance. Members of subfamily 2 probably participate in ion absorption (Rubio *et al.*, 1995; Horie *et al.*, 2001, 2007; Yao *et al.*, 2010), as indicated by their localization at the plasma membrane (Horie *et al.*, 2007, 2011; Lan *et al.*, 2010; Xue *et al.*, 2011).

In *A. thaliana*, only a single member of the HKT family, *At*HKT1;1, is present (Uozumi *et al.*, 2000), while the rice genome contains nine HKT genes, one of them probably a pseudo-gene (Garciadeblás *et al.*, 2003). The presence of more than one HKT gene in rice, with members from the two subfamilies, indicates that the corresponding proteins must play particular roles in the physiology of the plant, either by expression in a particular tissue or organelle, or by presenting unique transport properties. Previous studies have confirmed this view, with members from the two subfamilies showing differences in ion selectivity (Horie *et al.*, 2001, 2011; Golldack *et al.*, 2002; Jabnoune *et al.*, 2009; Yao *et al.*, 2010; Sassi *et al.*, 2012) and differential gene expression profiles in plant tissues (Golldack *et al.*, 2002; Kader *et al.*, 2006; Jabnoune *et al.*, 2009).

*Os*HKT1;3 functions as a highly selective Na^+^ transporter and its transcript is expressed in the vascular tissue of roots and leaves. The high expression in leaf adaxial epidermal bulliform cells has been used to propose its involvement in turgor changes for rolling and unrolling of leaves in response to environmental variations (Jabnoune *et al.*, 2009). Of particular interest is the high Na^+^ selectivity of *Os*HKT1;3 which, if not unique among HKTs, raises the question of the role of such a mechanism, in view of the toxic effects that sodium has on glycophytes, such as rice, and its widespread expression in the plant (Jabnoune *et al.*, 2009). To study the regulation of *Os*HKT1;3, interacting proteins that might regulate its activity/trafficking were searched for employing a large-scale protein–protein interaction screen based on the mating-based split ubiquitin system (mbSUS) (Lalonde *et al.*, 2010; Chen *et al.*, 2012; Jones *et al.*, 2014). In this screen, a plant homologue of the putative cargo receptor, cornichon, was identified. Here the characteristics of rice cornichon are reported at the cellular level, together with its interaction with *Os*HKT1;3 as a cargo protein, and the intracellular localization of *Os*HKT1;3 to the Golgi system where it may function as the shunt conductance for the H^+^ pumps that acidify this organelle.

## Materials and methods

### Mating-based split ubiquitin system (mbSUS)

For PCR amplification of the open reading frame (ORF) for *OsHKT1;3* and *OsCNIH1* genes without their stop codon, the primers described in Supplementary Table S1 available at *JXB* online were employed. Entry clones were generated with the pENTR-TOPO vector/plasmid following the manufacturer’s instructions (Invitrogen). The integrity of gene insertion was confirmed by sequencing and by digestion with *Pvu*II (Roche). The LR clonase (Invitrogen) was employed to transfer the *OsHKT1;3* and *OsCNIH1* genes to the pMETYC_GW (Cub clones) and pXN32_GW (Nub clones) vectors, respectively. Yeast media were prepared as previously described (Lalonde *et al.*, 2010). The THY.AP4 (*MATa ura3, leu2, lexA::LacZ::trp1 lexA::HIS3 lexA::ADE2*) and THY.AP5 (*MATα URA3*, *leu2*, *trp1*, *his3 loxP*::*ade2*) yeast strains were transformed with the pMETYC_GW and pXN32_GW vectors, respectively (Obrdlik *et al.*, 2004), employing the LiAc protocol previously described (Lalonde *et al.*, 2010). Cub clones were pre-screened to identify false-positive and false-negative fusions. Those fusions that did not interact with the soluble NubWT, that has a strong affinity for the Cub domain, were considered as false negatives; while those that did interact with the NubG (with reduced affinity for Cub) corresponded to false positives. Those Cub fusions that passed the pre-screening were used with the *A. thaliana* membrane-linked interactome protein library Nub clones. Positive interactors were chosen by the similar growth shown to the soluble NubWT in IS-500 medium (Lalonde *et al.*, 2010).

### Transitory expression of *Os*HKT1;3 and *Os*CNIH1 tagged with florescent proteins in *Nicotiana benthamiana* leaf epidermis

For the transitory transformation of *N. benthamiana* leaves, plants were grown in the glasshouse from seeds in pots containing Metromix 500 soil (SunGro) at 25 °C under natural light conditions. Leaves from 4-week-old plants were infiltrated with *Agrobacterium tumefaciens* harbouring either the p*OsHKT1;3*-EYFP, p*OsHKT1;3*-mCherry, or p*OsCNIH1*-mCherry constructs under the control of the 35S promoter and/or the indicated subcellular marker gene constructions (Nelson *et al.*, 2007). For bimolecular fluorescence complementarion (BiFC) experiments, leaf infiltration was done using the constructs *pYFC43-OsHKT1;3*, *pYFC43-OsCNIH1*, *pYFC43-AtPIP2A*, *pYFN43-OsHKT1;3*, *pYFN43-OsCNIH1*, and *pYFN43-AtPIP2A* (Belda-Palazón *et al.*, 2012). *Agrobacterium tumefaciens* containing each construct were grown in 30ml of LB with rifampicin (50 μg ml^–1^) and spectinomycin (50 μg ml^–1^) or kanamycin (5050 μg ml^–1^) at 28 °C at an OD_600_ between 0.3 and 0.5. Leaves were infiltrated with a solution containing sodium phosphate buffer pH 7, 0.1mM acetosyringone (Sigma), 28mM glucose, and bacterial culture with an OD_600_ of 0.3. To obtain the expression clone for each gene, an LR gateway-based recombination reaction (Invitrogen) with either pX-EYFP-GW or pX-Cherry-GW for C-terminal translational fusions, or pYFC43 and pYFN43 for BiFC assays, were achieved. Transformed electrocompetent cells of the strain GV3101 of *A. tumefaciens* with each of these constructions were used for expression in tobacco. Image J software (http://imagej.nih.gov/ij/) was used to analyse the confocal images for co-localization. Pearson’s coefficient and scatter plots were obtained using the JACoP Plug-in (Bolte and Cordelières, 2006).

### Functional expression of *Os*HKT1;3 in *Xenopus* oocytes

The two-electrode voltage-clamp technique was used to record the activity of *Os*HKT1;3 and *Os*CNIH1, individually or co-expressed in oocytes 2 d after cRNA injection as described (Ortiz-Ramirez *et al.*, 2011). For all measurements, oocytes were clamped at their free running membrane potential. The reported results are the means ±SD of 5–10 oocytes. For expressing *OsHKT1;3* and *OsCNIH1* in *Xenopus* oocytes, both genes were cloned into the pOO2 oocyte expression vector (Ludewig *et al.*, 2002) employing the primers described in Supplementary Table S1 at *JXB* online. An *Os*HKT1;3–EGFP fusion protein was made by adding the ORF of the enhanced green fluorescent protein (pEGFP-C1; Clontech) at the C-terminus of *OsHKT1;3* which was then subcloned into pOO2. Clones were verified by sequencing. Capped cRNA was transcribed *in vitro* by SP6 RNA polymerase using the mMessage mMachine kit (Ambion), after linearization of the plasmid with Bbr P1 (Roche).

### Heterologous expression of *Os*HKT1;3 in yeast

*OsHKT1;3* was amplified by PCR employing the primers described in Supplementary Table S1 at *JXB* online. The PCR-amplified *OsHKT1;3* gene was inserted into the YEp352-NHA1-URA and pGRU1-NHA1-URA vectors (previously digested with *Pst*I and *Pvu*II, respectively) behind the *Saccharomyces cerevisiae* NHA1 promoter (Kinclová *et al.*, 2001) using homologous recombination in *S. cerevisiae* BW31a cells (*MATa leu2-3/122 ura3-1 trp1-1 his3-11/15 ade2-1 can1-100 GAL SUC2 mal10 ena1-4Δ::HIS3 nha1::LEU2*). The resulting vectors were pYEp352-*OsHKT1;3* and pGRU1-*OsHKT1;3*. The correct constructions were confirmed by sequencing using oligos described in Supplementary Table S1. *OsCNIH1* and *ERV14* cloned in pENTR-TOPO were transferred to pDR-F1-GW-LEU by an LR reaction (Invitrogen). pDR-F1-OsCNIH1 and pDR-F1-ERV14 constructs are under control of the *PMA1* promoter. Constructions were verified by *Pvu*II digestion (Thermo Scientific). The *S. cerevisiae* BY4741 strain (*MATa his3Δleu2Δmet15Δura3Δ*) was used to determine NaCl sensitivity in drop-test assays. Yeast cells were grown aerobically at 30 °C in standard YPD (to prepare competent cells for transformation) or Yeast Nitrogen Base (YNB) media without amino acids (to select and maintain transformants), with 2% glucose as carbon source and appropriate auxotrophic supplements. The growth phenotype of cells was estimated in drop tests on solid YNB medium supplemented with NaCl after 4 d. The *ERV14* gene was deleted by homologous recombination using the KanMX marker gene and the Cre–loxP system-producing strain BY4741*Δerv14* (Supplementary Fig. S1). Primers used are described in Supplementary Table S2.

### Fluorescence microscopy

Fluorescence from EYFP was visualized by excitation with an Argon laser at 514nm with the spectral detector set between 540nm and 30nm for the emission. The wavelengths employed for Citrine and mCherry were 488nm and 543nm (excitation), and 515/30nm and 630/60nm (emission), respectively. Abaxial epidermal peels of mature leaves were placed onto microscope slides in water, covered with a cover slide, and observed by fluorescence microscopy using an inverted multiphoton confocal microscope (Olympus FV1000) equipped with a ×60 oil immersion objective. Results are representative images from >10 cells from at least four different independent transformations.

## Results

### Analysis of protein–protein interactions between *Os*HKT1;3 and the *Arabidopsis* membrane interactome

In order to obtain additional information on the functioning of *Os*HKT1;3, the possible regulation of the transporter through protein–protein interactions was studied by employing the mbSUS (Obrdlik *et al.*, 2004; Lalonde *et al.*, 2010). Screening an *A. thaliana* membrane-linked interactome protein library as prey (Nub clones), with *Os*HKT1;3 protein used as bait (Cub clone) (Lalonde *et al.*, 2010; Jones *et al.*, 2014), 19 possible interactions were identified, as indicated by growth of diploid yeast cells under selective conditions (Table 1). These interactions were common to two other rice HKTs, *Os*HKT1;1 and *Os*HKT2;1 (Table 1).

**Table 1.** Arabidopsis *proteins interacting with* Os*HKT1;3* *Arabidopsis* proteins identified with the mbSUS employing *Os*HKT1;3 as bait (Cub clone) and the *Arabidopsis* interactome (Nub clones) (Jones *et al.*, 2014) as prey.

*Os*HKT1;3 (Cub)	AGs *Arabidopsis* (Nub)	Description
Os02g07830	AT3G17000	Ubiquitin-conjugating enzyme 32
	AT3G25805	Unknown protein
	AT1G17280	Ubiquitin-conjugating enzyme 34
	AT1G21240	Cell wall-associated kinase
	AT1G34640	Unknown protein
	AT1G47640	Unknown protein
	AT1G63110	Cell division cycle protein-related
	AT2G26180	IQ-domain 6 (IQD6)
	AT3G08040	Member of the MATE family
	AT3G10640	VPS60 vesicle-mediated transport
	AT3G12180	Cornichon family protein
	AT3G28220	Meprin and TRAF homology domain-containing protein
	AT4G30850	Heptahelical transmembrane protein homologous to human adiponectin receptors and progestin receptors
	AT5G06100	myb family of transcription factors (MYB33)
	AT5G06320	Tobacco hairpin-induced gene (HIN1)
	AT5G10450	14-3-3 gene family
	AT5G37050	Unknown protein
	AT5G49540	Unknown protein
	AT5G52240	Similar to progesterone-binding proteins in animals.

### *Os*HKT1;3 interacts with *Os*CNIH1 in yeast

**Fig. 1.**
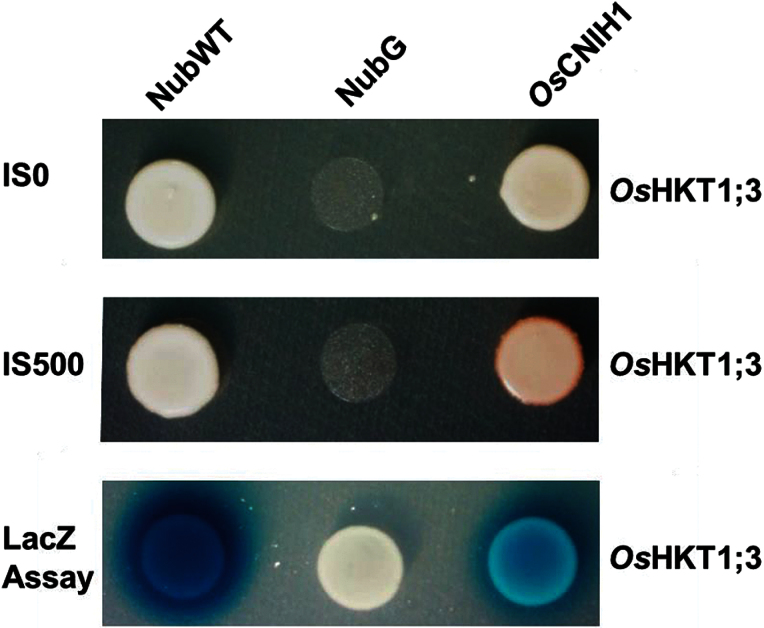
Homologous interaction between *Os*HKT1;3 and *Os*CNIH1. Homologous interaction between *Os*HKT1;3 and *Os*CNIH1 confirmed with the mbSUS in yeast with selective medium in the absence (IS0) or in the presence of methionine (0.5mM; IS500). Corroboration of the interaction between *Os*HKT1;3 and *Os*CNIH1 was demonstrated by activation of LacZ and revealed with X-Gal as a substrate. Representative results of three different assays are shown.

After analysing the 19 interacting proteins, it was decided to focus on a protein homologous to Erv14p in yeast and cornichon in *Drosophila* and mammals, which has been shown to direct the trafficking of membrane proteins from the ER to the Golgi apparatus (Gillingham *et al.*, 2004), or to the plasma membrane (Schwenk *et al.*, 2009; Herzig *et al.*, 2012). Confirmation of the interaction between *Os*HKT1;3 and rice cornichon (*Os*CNIH1; Os06g04500) was obtained by employing *OsHKT1;3* and *OsCNIH1* as Cub and Nub clones, respectively, in the mbSUS. Growth of diploid yeast cells in selective medium confirmed the interaction between *Os*HKT1;3 and *Os*CNIH1 (Fig. 1; IS0), and the strength of the interaction was demonstrated by growth of the diploid cells in the presence of 0.5mM methionine which acts as a repressor of the pMETYC promoter (Grefen *et al.*, 2007; Fig. 1, IS500). As expected, *Os*HKT1;3 did not interact with soluble NubG, but it did with soluble NubWT, that were used as false-positive and false-negative controls, respectively (Fig. 1). Additional confirmation of the interaction between *Os*HKT1;3 and *Os*CNIH1 was derived from analysing LacZ activity with X-Gal as a substrate, whereby clear blue signals in the diploid cells harbouring *Os*HKT1;3 and *Os*CNIH1 clones were observed. The blue precipitate was also observed with the positive control (soluble NubWT), but not with soluble NubG, the negative control (Fig. 1, LacZ).

### Sequence analysis of rice cornichon

**Fig. 2.**
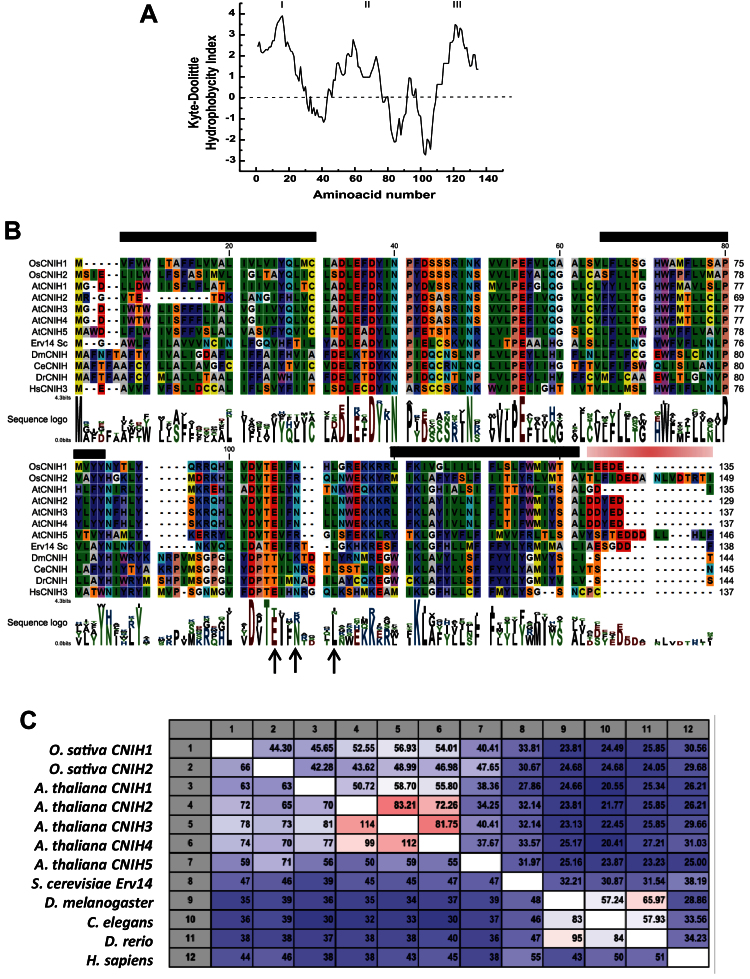
*Os*CNIH1 sequence analysis. (A) A Kyte–Doolittle hydropathy plot shows three potential transmembrane domains (I, II, and III; values >0) in *Os*CNIH1. (B) Sequence alignment of *Os*CNIH1 with *Os*CNIH2, *At*CNIH1, *At*CNIH2, *At*CNIH3 *At*CNIH4, *At*CNIH5, *Sc*Erv14, and cornichon homologues from *Drosophila* (*Drosophila melanogaster*), worm (*Caenorhabditis elegans*), zebra fish (*Danio rerio*), and human isoform 4 (*Homo sapiens*). Accession numbers are *Os*06g04500, *Os*12g32180, At4g12090, At1g12340, At1g12390, At1g62880, At3g12180, YGL054C Erv14, NP_477068, CAB01516, NP_001028278, and NP_001264129.1, respectively. Black bars indicate putative transmembrane domains; arrows indicate conserved residues involved in binding to COPII in yeast (I96, F97, and L100); the grey bar denotes an acidic domain. (C) Pairwise comparison between the cornichon proteins listed in (B) shows the percentage identity (upper right values) and number of identical residues (bottom left values). (This figure is available in colour at *JXB* online.)

The rice genome contains two cornichons, *Os*CNIH1 (*Os*06g04500), a small hydrophobic protein of 135 amino acids (Fig. 2A, B), and *Os*CNIH2; (*Os*12g32180), which encodes a slightly larger protein (149 amino acids; Fig. 2B). The two isoforms share 44% identity at the amino acid level. Cornichon belongs to a family of membrane proteins unique to eukaryotes that is predicted to have three membrane-spanning α-helixes (Fig. 2A, Kyte–Doolittle; Fig. 2B, TMHMM, black lines), with the N-terminus towards the cytoplasm according to the predicted positive-inside rule (von Heijne, 1992), and the C-terminus located in the ER lumen. All plant cornichon homologues possess an acidic domain in the luminal C-terminus (Fig. 2B, red line). The similarity between plants, yeast, fly, worm, zebra fish, and human cornichons is ~40%, with *Os*CNIH1 showing higher identity with the *Arabidopsis* homologues *At*CNIH2, *At*CNIH3, and *At*CNIH4 (Fig. 2C).

### Split-YFP analyses demonstrate that *Os*HKT1;3 and *Os*CNIH1 interact in the ER

**Fig. 3.**
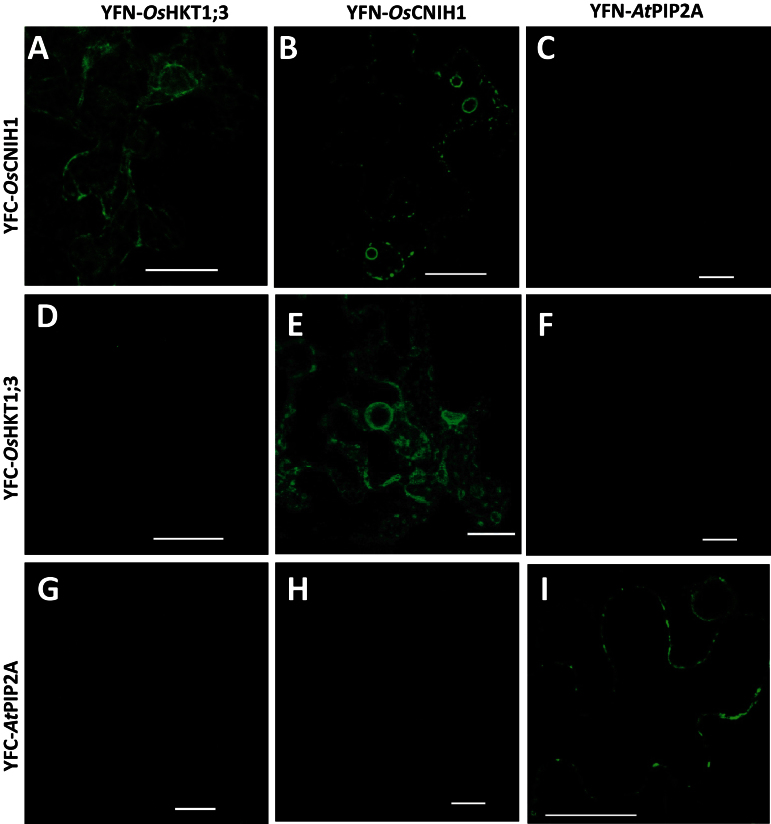
BiFC confirms the interaction of *Os*CNIH1 with *Os*HKT1;3 and demonstrates the likely oligomerization of *Os*CNIH1 in transiently transfected tobacco leaves. (A, E) Reciprocal interaction between YFC–*Os*CNIH1/YFN–*Os*HKT1;3 and YFN–*Os*CNIH1/YFC–*Os*HKT1;3 confirms the interaction of the two proteins in the ER. (B) Co-expression of YFC–*Os*CNIH1 and YFN–-*Os*CNIH1 indicates the possible oligomerization of cornichon in the ER. Absence of a fluorescence complementation signal indicates that: *Os*CNIH1 does not interact with *At*PIP2A (C, H); *Os*HKT1;3 does not form oligomers (D); and the transporter and the aquaporin do not interact (F, G). (I) Oligomerization of *At*PIP2A. Scale bar=25 μm.

To confirm the interaction between *Os*HKT1;3 and *Os*CNIH1 *in planta*, and to identify the site(s) of interaction, the split-YFP (yellow fluorescent protein) system was employed by fusing the N-terminal half of *EYFP* to the N-terminal half of *OsHKT1;3* (*YFN-OsHKT1;3*) and the C-terminus of *EYFP* to the N-terminus of *OsCNIH1* (*YFC-OsCNIH1*). Co-expressing these constructs in tobacco leaves led to fluorescence recovery of EYFP (Fig. 3A), indicating the association between *Os*HKT1;3 and *Os*CNIH1 at the ER, according to the reticulated structure highlighted by EYFP. The co-localization of the two proteins was confirmed with fusions of the N- and C-terminal halves of EYFP to the N-terminus of *Os*CNIH1 (YFN–*Os*CNIH1) or *Os*HKT1;3 (YFC–*Os*HKT1;3), respectively (Fig. 3E). As a positive control for the BiFC assay, the well-known tetramerization of the plasma membrane aquaporin *At*PIP2A was used. For this, the N- or C-terminal halves of EYFP were fused to the N-terminus of the aquaporin *At*PIP2A (YFN–*At*PIP2A and YFC–*At*PIP2A). As expected, oligomerization of the aquaporin was observed at the plasma membrane (Fig. 3I). Co-expression of *YFN-OsCNIH1* and *YFC-AtPIP2A* did not lead to reconstitution of EYFP fluorescence (Fig. 3C, H). Additional analyses demonstrated fluorescence reconstitution by expressing the C- and N-termini of YFP fused independently to the N-terminus of *Os*CNIH1, indicating the oligomerization of the protein in intracellular structures that resembled the Golgi apparatus (Fig. 3B). In contrast, similar studies with *Os*HKT1;3 failed in reconstituting YFP fluorescence, suggesting that this transporter does not oligomerize (Fig. 3D). No interactions were observed between the aquaporin *At*PIP2A and *Os*HKT1;3 or *Os*CNIH1 (Fig. 3C, F–H). These results demonstrated that the interaction between *Os*HKT1;3 and *Os*CNIH1 occurs at the ER, and indicated the possible oligomerization of rice cornichon. Moreover, the results suggested that BiFC did not seem to be a result of overexpression of the proteins in the same membrane, as indicated by failure in reconstituting EYFP associated with *Os*HKT1;3.

### Cornichon co-localizes with *Os*HKT1;3 and resides in the ER and Golgi

**Fig. 4.**
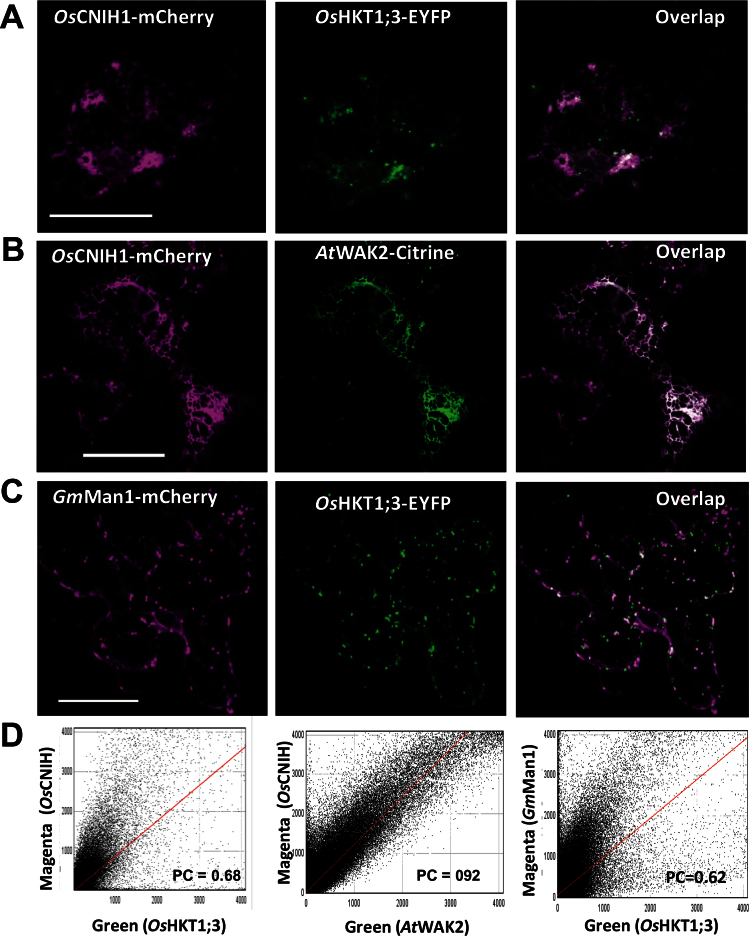
Intracellular co-localization of *Os*CNIH1 with *Os*HKT1;3 in plants. (A) Expression of *Os*CNIH1–mCherry (left) and *Os*HKT1;3–EYFP (centre), and co-localization of the two proteins (right) in tobacco leaves. (B) Expression of *Os*CNIH1–mCherry (left) and the ER marker *At*WAK2–Citrine (centre), and co-localization of the two proteins (right). (C) Expression of *Os*HKT1;3–EYFP (centre) and the Golgi marker *Gm*Man1–mCherry (left), and co-localization of the two proteins (right). (D) Scatter plots of pixel distribution of the magenta (*y*-axis) and green (*y*-axis) channels employing the Costes algorithm for images shown in (A, left), (B, centre), and (C, right). Scale bar=25 μm.

Further support for the interaction between *Os*HKT1;3 and *Os*CNIH1 was obtained by analysing tobacco leaf epidermis co-transformed with *OsCNIH1-mCherry* and *OsHKT1;3-EYFP*. Expression of *Os*CNIH1–mCherry was generally observed as a reticulated structure (Fig. 4A, left), while that of *Os*HKT1;3–EYFP mainly appeared as bright puncta (Fig. 4A, centre). Overlapping both images showed that the puncta associated with *Os*HKT1;3–EYFP superimposed the reticula highlighted by *Os*CNIH1–mCherry (Fig. 4A, right, white dots). By plotting the pixel number for *Os*HKT1;3–EYFP against that from *Os*CNIH1–mCherry, pixel distribution was demonstrated to occur along a straight line (Fig. 4D, left), and, by applying Costes’ method (Costes *et al.*, 2004) for image analysis using ImageJ (JACoP Plug-in), a Pearson’s coefficient (PC) and *P*-value of 0.68 and 100%, respectively, were calculated (Fig. 4D, left) (Bolte and Cordelières, 2006). These findings confirmed that *Os*CNIH1 and *Os*HKT1;3 partially co-localized in the ER. In view of these results, it was important to determine and confirm the site(s) of residence for *Os*CNIH1 and *Os*HKT1;3 individually, and for this co-expression studies were carried out employing several well-characterized membrane markers fused to fluorescent proteins. *Os*CNIH1–mCherry highlighted a reticulated structure, strongly suggesting its expression at the ER (Fig. 4B, left); this was confirmed by the comparable expression observed for the ER marker *At*WAK2–Citrine (Fig. 4B, centre) (Nelson *et al.*, 2007) and by the overlapping of signals from these two markers (Fig. 4B, right, white signal). Image analysis demonstrated a high degree of co-localization between *Os*CNIH1 and *At*WAK2 (PC=0.82; *P*=100%; Fig. 4D, centre). Localization of *Os*HKT1;3–EYFP emphasized small, highly motile punctate structures in the cytoplasm, indicating localization to potentially vesicular structures (Fig. 4C, centre). To identify this compartment, *Os*HKT1;3–EYFP was co-expressed with the Golgi marker *Gm*Man1–mCherry (Nelson *et al.*, 2007), and similar punctate cytoplasmic signals were observed for both proteins (Fig. 4C, left and centre), that when overlapped showed partial co-localization (Fig. 4C, right, white areas). Applying Costes’ method, a calculated PC of 0.62 with a *P*- value of 100% was obtained, confirming the co-localization of the two proteins (Fig. 4D, right) which indicated that *Os*HKT1;3–EYFP partially localized to the Golgi.

**Fig. 5.**
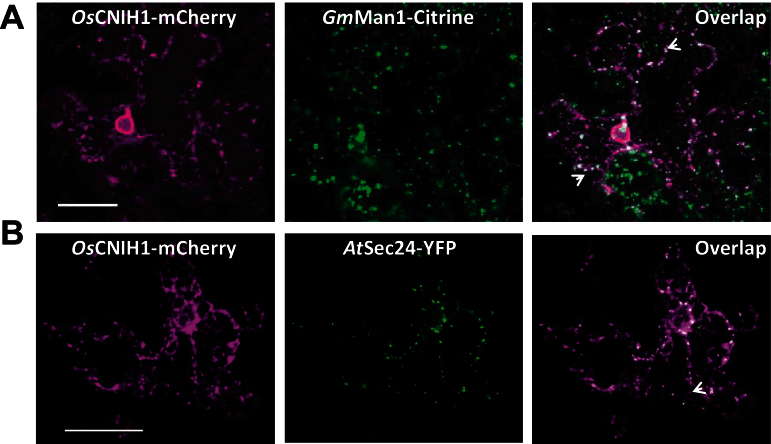
*Os*CNIH1 is also located at the Golgi and ERES. (A) Expression of *Os*CNIH1–mCherry (left) and the Golgi marker *Gm*Man1–Citrine (centre), and the co-localization of the two proteins (right). (B) Expression of *Os*CNIH1–mCherry (left) and the ERES/COPII marker *At*Sec24–YFP (centre), and overlapping of the two images showing the co-localization of the two proteins (right). Arrows indicate the punctate sites of co-localization (white signal). Scale bar=25 μm.

Work in yeast and mammals hase demonstrated that cornichon homologues did not locate exclusively to one specific compartment, but rather circulated actively between the Golgi and the ER, in association with COPII vesicles (Powers and Barlowe, 1998; Harmel *et al.*, 2012). To identify more clearly the plant cornichon location, *Gm*Man1–Citrine was used as a Golgi marker and *At*Sec24–YFP as a marker for ER exit sites (ERES)/COPII (Hanton *et al.*, 2009; Langhans *et al.*, 2012). Co-expression of *Os*CNIH1–mCherry (Fig. 5A, left) with *Gm*Man1–Citrine (Fig. 5A, centre) showed that both proteins highlighted punctate structures with properties similar to that of the Golgi, with *Os*CNIH1–mCherry also highlighting the network associated with the ER, particularly surrounding the nucleus (Fig. 5A, left). Quantitative analyses of the overlapped images (Fig. 5A, right) showed a relatively low correlation (PC=0.40, *P*=100%; Supplementary Fig. S2A at *JXB* online), indicating that *Os*CNIH1 was also present in the Golgi apparatus. When *Os*CNIH1 was co-expressed with *At*Sec24–YFP, *Os*CNIH1 labelled the ER (Fig. 5B, left), while *At*Sec24 appeared as bright puncta dispersed throughout the cytoplasm (Fig. 5B, centre). *At*Sec24–YFP co-localized with the reticulate *Os*CNIH1–mCherry fluorescence (Fig. 5B, right) as indicated by the calculated PC=0.66 and *P*=100% (Supplementary Fig. S2B), corresponding to the proposed structure for the ERES/COPII compartment. Together, these imaging data indicated that rice cornichon located to the Golgi and ER.

### Subcellular localization of *Os*HKT1;3

**Fig. 6.**
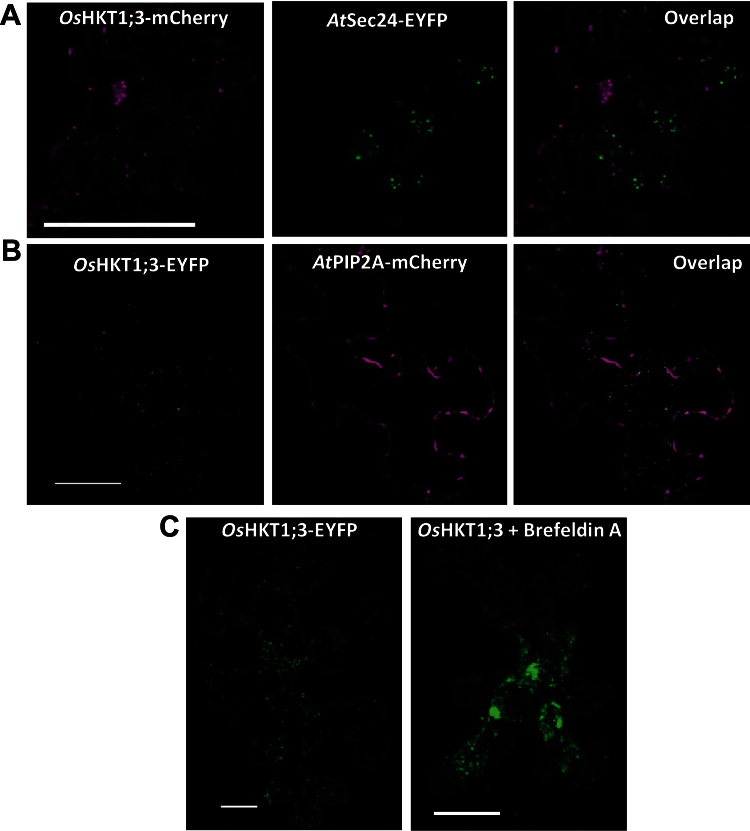
*Os*HKT1;3 does not localize to the ERES or the plasma membrane and forms aggregates upon exposure to brefeldin A. (A) Expression of *Os*HKT1;3–mCherry (left) and the ERES/COPII marker *At*Sec24–EYFP (centre), and overlapping of the two images (right). (B) Co-localization analysis of *Os*HKT1;3–EYFP (left) with the plasma membrane marker *At*PIP2A–mCherry (centre), and overlapping of the two images (right). The intracellular localization of *Os*HKT1;3–EYFP, seen as fluorescent puncta distributed throughout the cell (C, left), was modified after incubation of the epidermis with brefeldin A at 25 μM for 15min, resulting in the formation of aggregated bodies (C, right). Scale bar=25 μm.

The intracellular localization of *Os*HKT1;3 (Fig. 4A, C) differed from that of plasma membrane-localized HKT isoforms (Horie *et al.*., 2007, 2011; Lan *et al.*, 2010; Xue *et al.*, 2011). To explore the localization of *Os*HKT1;3 in more detail, co-localization analysis was performed with the same membrane markers used for *Os*CNIH1 (Figs 4, 5). Co-expression of *Os*HKT1;3–mCherry with *At*Sec24–YFP demonstrated that both proteins were visualized as punctate structures that did not locate to the same compartment (Fig. 6A). Confirmation that co-localization of *Os*HKT1;3–mCherry and *At*Sec24–YFP was low (Fig. 6A, right) was supported by the low PC of 0.32 (*P*=100%; Supplementary Fig. S2C at *JXB* online). Co-expression of *Os*HKT1;3 and *At*PIP2A–mCherry in tobacco leaf epidermal cells verified that the puncta corresponding to *Os*HKT1;3–EYFP localized to the cytoplasm (Fig. 6B, left), while expression of *At*PIP2A–mCherry was limited to the plasma membrane (Fig. 6B, centre). Merging of the two channels yielded few co-localization points (Fig. 6B, right); quantitative analysis revealed no co-localization between *Os*HKT1;3 and *At*PIP2A, as indicated by the low PC of 0.14 (*P*=100%; Supplementary Fig. S2D). Additional evidence supporting the Golgi as the site of residence for *Os*HKT1;3 was obtained with use of the fungal toxin brefeldin A (BFA). BFA blocks the activation of the GTPase Arf1 by causing a non-productive complex with its Sec7 GTP exchange factor (Chardin and McCormick, 1999), resulting in the formation of Golgi aggregates described as BFA bodies (Ritzenthaler *et al.*, 2002). Incubation of tobacco leaf epidermis transformed with *OsHKT1;3-EYFP* for 15min with 25 μM BFA caused the appearance of relatively large fluorescent aggregates (Fig. 6C, right) that resembled BFA bodies, in comparison with the smaller puncta from *Os*HKT1;3–EYFP in the untreated epidermis (Fig. 6C, left). Measurement of the rapid movement of the fluorescent puncta associated with *Os*HKT1;3–EYFP gave a mean velocity of 0.14±0.02 μm s^–1^ (mean ±SD, *n*=9; Supplementary Fig. S3, Supplementary Video S1), similar to the rates that have been reported for the highly motile Golgi apparatus (Boevink *et al.*, 1998). Although some puncta did not move, corresponding to stationary Golgi stacks (Lerich *et al.*, 2012), others moved at a lower speed that varied between 0.081 μm s^–1^ and 0.061 μm s^–1^. All these observations provided further evidence that *Os*HKT1;3–EYFP locates to the Golgi apparatus of plant cells.

### Co-expression of *Os*CNIH1 and *Os*HKT1;3 in *Xenopus* oocytes modified the cellular localization of the transporter and inhibited its activity

**Fig. 7.**
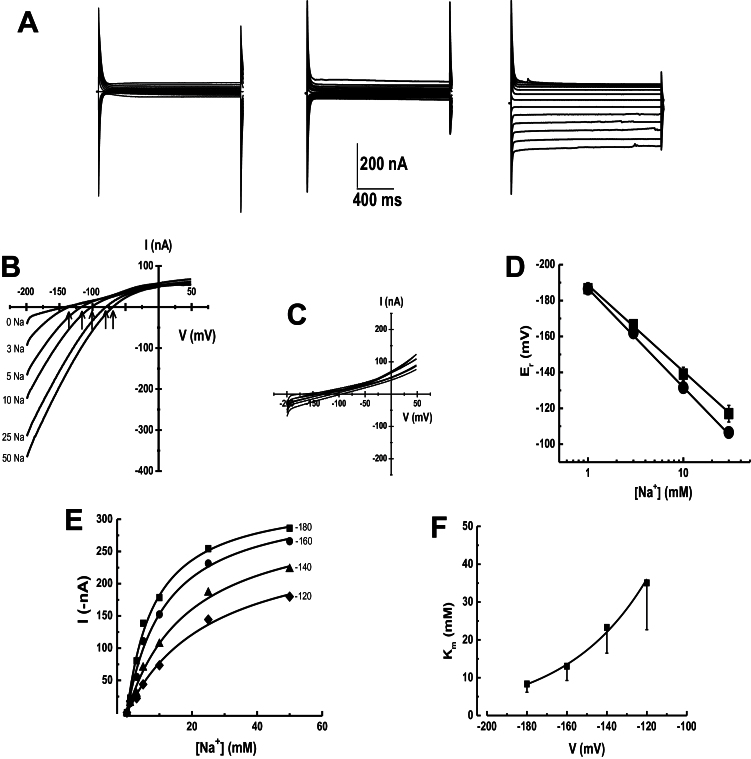
Transport properties of *Os*HKT1;3. (A) Original traces of currents activated by voltage pulses between –200 mV and 50 mV in 20 mV steps from a control water-injected oocyte exposed to 30mM NaCl (left), or expressing *Os*HKT1;3 exposed to the bath solution either without (centre) or with 30mM NaCl (right). (B) I–V plot from currents recorded in an oocyte expressing *Os*HKT1;3 and exposed to different concentrations of NaCl (mM); *E*_r_ (arrows). (C) I–V plot from currents recorded from a control oocyte and exposed to the same NaCl solutions as in (B). (D) Plot showing the linear relationship between *E*_r_ and extracellular Na^+^ concentrations from oocytes expressing *Os*HKT1;3 in the absence (circles) or presence of 1mM KCl (squares). Lines are least square linear regressions fits with a slope of 54.2 mV per decade. (E) Sodium transport kinetics were voltage dependent. Lines are fits to the Michaelis–Menten equation at the corresponding voltages with *r* ^2^ ≥0.9. (F) The affinity (*K*_m_) of *Os*HKT1;3 for Na^+^ was voltage dependent. The line is a fit to Equation 1. Data are from more than five oocytes from 3–4 different frogs and correspond to the mean ±SD.

In view of the results that indicated the interaction and co-localization of *Os*CNIH1 and *Os*HKT1;3 in plant cells, the oocyte expression system was employed to study the potential effects of *Os*CNIH1 on the transport activity of *Os*HKT1;3. Initially, the properties of the transporter reported as an Na^+^-selective transport mechanism were confirmed (Jabnoune *et al.*, 2009). Oocytes injected with *OsHKT1;3* cRNA showed the activation of inward currents when exposed to Na^+^ solutions (Fig. 7A, right), but not when the cation was absent (Fig. 7A, centre). A control water-injected oocyte exposed to sodium failed to activate any measurable currents (Fig. 7A, left). Confirmation that the inward currents corresponded to Na^+^ movement was derived from the increasing current magnitude recorded with increasing concentrations of Na^+^, together with clear shifts in the reversal potential (*E*_r_) (Fig. 7B, arrows) activated by voltage ramps. Similar experiments with a water-injected oocyte did not show changes in current magnitude or in *E*_r_ with variable Na^+^ concentrations (Fig. 7C). Plotting the *E*_r_ values against external Na^+^ concentrations gave a linear relationship with a slope of 54.2 mV per decade Na^+^ (Fig. 7D, circles). A similar relationship was observed for *E*_r_ values obtained in the presence of 1mM K^+^ (Fig. 7D, squares). These results clearly indicated that *Os*HKT1;3 functions as an Na^+^-selective transporter/channel and that *Os*HKT1;3 does not function as an Na^+^/K^+^ symporter, as has been reported for some members of the subfamily 2 of HKT transporters (Rubio *et al.*, 1995; Gassmann *et al.*, 1996; Horie *et al.*, 2001). Kinetic properties of the transporter were obtained by plotting current magnitude from voltage ramps against external Na^+^ concentrations at different holding potentials, observing saturation at concentrations >50mM (Fig. 7E). Data analysis with the Michaelis–Menten equation showed that the apparent transport affinity constant (*K*_m_) for Na^+^ increased at less negative holding potentials, with values of 6.4, 9.6, 17.1, and 42.6mM obtained at –180, –160, –140, and –120 mV, respectively (Fig. 7E). Assuming a single binding site for sodium, Equation 1 was employed to evaluate the voltage dependence of *K*_m_, where δ is the fractional electrical distance, *e* is the elementary charge, *V* is the membrane potential, *k* is Boltzmann’s constant, and *T* is the absolute temperature (Woodhull, 1973).

$$K_{m}\left(\delta \right)\;\;=\;K_{m}^{\left(0\;mV\right)}*exp(\delta *e*\frac{V}{k*T})$$ (1)

**Fig. 8.**
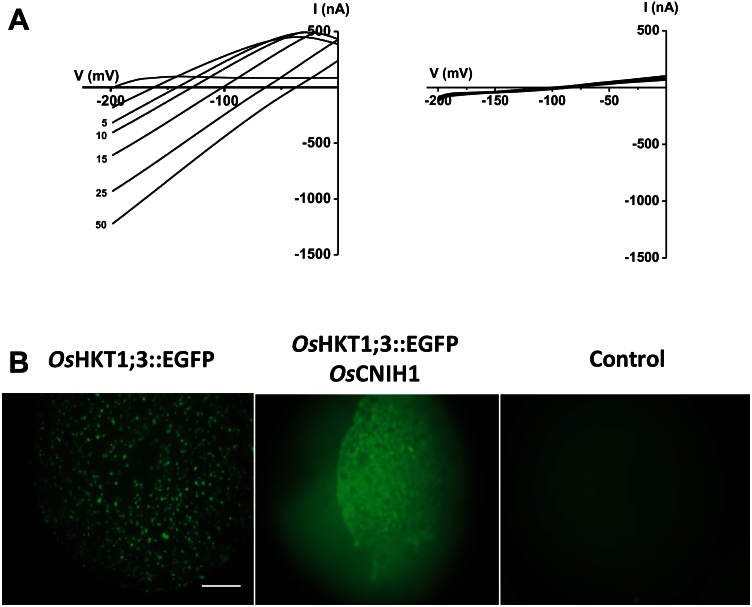
Co-expression of *Os*HKT1;3 and *Os*CNIH1 in *Xenopus* oocytes caused retention of the transporter in the ER, preventing the activation of Na^+^ currents. (A) Sodium inward currents activated by voltage ramps from an oocyte expressing *Os*HKT1;3 (left). Co-expression of *Os*HKT1;3 and *Os*CNIH1 (cRNA ratio injected 1:1) in a *Xenopus* oocyte (right). (B) Expression of *Os*HKT1;3–EGFP was observed as puncta at the plasma membrane of a *Xenopus* oocyte (left); upon co-expression with *Os*CNIH1, fluorescence was observed exclusively in a reticulated structure (centre). Autofluorescence is from a water-injected oocyte (right). Scale bar=250 μm. (This figure is available in colour at *JXB* online.)

From this analysis, the putative Na^+^ binding site was calculated to be located at (δ) 65% within the membrane electrical field (Fig. 7F, fitted line). Having confirmed the basic properties of *Os*HKT1;3 (Jabnoune *et al.*, 2009), the effects of co-expressing *Os*HKT1;3 together with *Os*CNIH1 in the oocyte system were then analysed. Voltage-clamp recordings showed that the activity of the transporter was inhibited by the presence of *Os*CNIH1, as indicated by the absence of inward currents at all extracellular Na^+^ concentrations tested (Fig. 8A, right). In comparison, an oocyte injected with only the cRNA for *OsHKT1;3*, activated large inward currents in response to voltage ramps, similar to those previously observed (Fig. 8A, left; see also Fig. 7D). The inhibition of *Os*HKT1;3 by co-injection of *Os*CNIH1 in the oocytes could be the result either of direct inhibition of the transporter activity or of the interaction between the two proteins preventing the default targeting of *Os*HKT1;3 to the oocyte membrane. To distinguish between these two possibilities, *EGFP* was fused to the C-terminus of *OsHKT1;3* and then the construct was expressed in albino *Xenopus* oocytes, individually or together with *OsCNIH1*, and the expression was observed under epifluorescence microscopy. When expressed alone, *Os*HKT1;3–EGFP was observed as punctate structures at the membrane of the oocyte (Fig. 8B, left). In contrast, when co-injected with *OsCNIH1*, *Os*HKT1;3–EGFP fluorescence was observed in the interior of the oocyte as a reticulated structure, resembling the ER (Fig. 8B, centre). Autofluorescence from a control water-injected oocyte was minimal (Fig. 8B, right).

### Deletion of *ERV14* in yeast modifies the intracellular location of *Os*HKT1;3 which is restored upon expression of *OsCNIH1* or *ERV14*

**Fig. 9.**
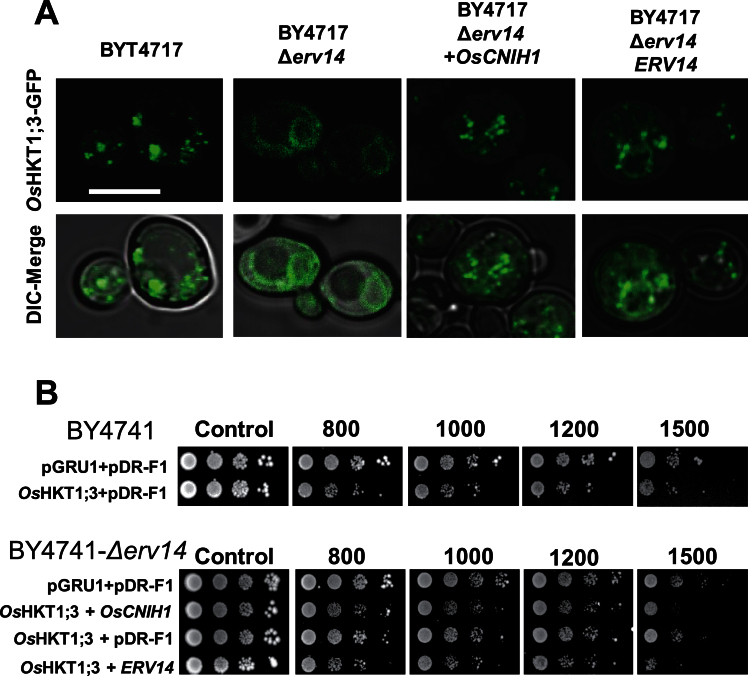
*Os*CNIH1 restores the intracellular expression of *Os*HKT1;3 in the yeast mutant BY4741*Δerv14* and sensitivity to NaCl. (A) Fluorescence and DIC images of living BY4741 and BY4741*Δerv14* cells expressing *Os*HKT1;3–GFP observed by confocal fluorescence microscopy and co-expressing either *OsCNIH1* or *ERV14p*. Scale bar=5 μm. (B) Drop-test assay on yeast strains BYT4741 (top) and BYT45*Δerv14* (bottom) grown in YNB solid medium with different Na^+^ concentrations and transformed with *pGRU1* and *pDR-F1*, *OsHKT1;3* and *pDR-F1*, *OsHKT1;3* and *OsCNIH1*, or *OsHKT1;3 and ERV14.* Representative results of at least three different experiments are shown.

To gain further insight into the interaction between *Os*CNIH1 and *Os*HKT1;3, the effect of mutating *ERV14*, the yeast cornichon homologue, on the heterologous expression of *Os*HKT1;3 tagged with GFP (*pGRU1-OsHKT1;3*) in BY4741 yeast cells was tested. Transformation of BY4741 yeast cells with the *pGRU1-OsHKT1;3* vector showed that localization of *Os*HKT1;3 was intracellular, highlighting several round bodies that indicated the presence of the transporter at the Golgi apparatus (Fig. 9A; BY4741) (Losev *et al.*, 2006; Matsuura-Tokita *et al.*, 2006). A different localization for *Os*HKT1;3–GFP was observed in the BY4741*Δerv14* mutant cells, as indicated by the diffused fluorescence observed around the nucleus and throughout most of the cytoplasm, a distribution that has been associated with the ER (Fig. 9A; BY4741*Δerv14*) (Manford *et al.*, 2012). Confirmation that localization of *Os*HKT1;3 at the Golgi apparatus depended on the presence of *Os*CNIH1 was obtained by co-transforming BY4741*Δerv14* cells with *OsCNIH1* and *OsHKT1;3-GFP* and observing the localization of the latter as round structures corresponding to the Golgi apparatus (Fig. 9A; BY4741*Δerv14+OsCNIH1*). Similar results were observed when rice cornichon was replaced with *ERV14* in the co-transformation of the BY4741*Δerv14* cells (Fig. 9A; BY4741*Δerv14+ERV14*), indicating that both rice cornichon and yeast Erv14p can direct the transporter to the Golgi apparatus. Additional evidence was gathered by investigating the effects of expressing *OsHKT1;3* in BY4741 and BY4741*Δerv14* yeast cells upon salt stress. Figure 9B (top) shows that transformation of BY4741 cells with *OsHKT1;3* and the empty *pDR-F1* vector (lower row) reduced cell growth between 0.8M and 1.5M NaCl, when compared with the parental cells transformed with the two empty vectors *pGRU1* and *pDR-F1* (Fig. 9B, upper row). Co-transformation of BY4741*Δerv14* cells with *OsHKT1;3* and the empty vector *pDR-F1*, in contrast, did not affect cell growth in the presence of NaCl (Fig. 9B, bottom, third row). Co-transformation of the BY4741*Δerv14* mutant cells with *OsCNIH1* and *OsHKT1;3* partially restored the sensitivity of the cells towards NaCl (Fig. 9B, bottom, second row), a similar response to that caused by the co-transformation of the BY4741*Δerv14* cells with *OsHKT1;3* and *ERV14* (Fig. 9B, bottom, fourth row).

## Discussion

### The putative cargo receptor *Os*CNIH1 interacts with the Na^+^ transporter *Os*HKT1;3

Protein–protein interactions play an essential role in cell structure and function, and have been shown to be important for protein function, regulation, and targeting (Lee *et al.*, 2009; Geiger *et al.*, 2010). The mbSUS with the *Arabidopsis* interactome (Lalonde *et al.*, 2010; Chen *et al.*, 2012; Jones *et al.*, 2014) was employed to identify proteins that might modulate the activity of *Os*HKT1;3 by direct protein–protein interactions. A total of 19 potential membrane protein interactions from *Arabidopsis* were identified (Table 1), and, from these, a protein which shows homology to *Drosophila* and human cornichon (CNIH) (Bökel *et al.*, 2006; Schwenk *et al.*, 2009) and yeast Erv14p (Powers and Barlowe, 1998, 2002) was selected for further analysis. In all these biological systems, the cornichon homologues have been described as cargo receptors for membrane proteins that may (Bökel *et al.*, 2006; Schwenk *et al.*, 2009) or may not (Powers and Barlowe, 1998, 2002; Herzig *et al.*, 2012) remain attached to the cargo. However, as far as could be assessed from the literature, there is no knowledge of the cornichon homologues in plants.

The interaction between the rice homologue *Os*CNIH1 and the Golgi membrane Na^+^ transport protein *Os*HKT1;3 was identified. Evidence for the interaction between *Os*HKT1;3 and *Os*CNIH1 was derived from growth of diploid yeast cells on selective media (IS0 and IS500) and activation of the reporter LacZ (Fig. 1). This interaction was confirmed *in planta* by bimolecular complementation of EYFP in tobacco cells where intracellular fluorescence signals were observed from reticulated structures reminiscent of the ER (Fig. 3A, E). This result was observed with either the C- or N-terminus of EYFP fused to the N-terminus of either *Os*CNIH1 or *Os*HKT1;3 (Fig. 3A, E). These observations were corroborated by co-localization studies between *Os*CNIH1 and *Os*HKT1;3 which also demonstrated the intracellular occurrence of both proteins in co-transformed tobacco leaves, where *Os*HKT1;3 was observed as small puncta superimposed on the ER highlighted by *Os*CNIH1 (Fig. 4A). Together, these results may be used to propose that direct interaction between *Os*CNIH1 and *Os*HKT1;3 occurs at the ER (Fig. 3A, E) and is required to direct the Na^+^ transporter to the Golgi (Fig. 4C). The absence of fluorescence complementation when tobacco leaves expressed the YFN–*Os*CNIH1 and YFN–*At*PIP2A protein chimeras indicated that the aquaporin does not interact with rice cornichon (Fig. 3C), and suggests that the interaction observed between *Os*CNIH1 and *Os*HKT1;3 was specific and not a general occurrence between cornichon and membrane proteins. Moreover, lack of EYFP fluorescence complementation by co-expression of YFN–*Os*HKT1;3 and YFC–*Os*HKT1;3 (Fig. 4D) indicates that overexpression of the protein at the same membrane does not, by itself, lead to reconstitution of the fluorescent protein, discarding the possibility that the results obtained with the interaction between *Os*CNIH1 and *Os*HKT1;3 are an artefact. This result was also supported by the absence of interaction observed with the mbSUS that indicated that *Os*HKT1;3 does not oligomerize (Supplementary Fig. S4 at *JXB* online). Corroboration of the interaction of *Os*CNIH1 with *Os*HKT1;3 was obtained independently by employing *Xenopus* oocytes co-expressing the two proteins, resulting in the retention of the transporter in internal structures (Fig. 8B, centre), and the inability to measure *Os*HKT1;3-dependent Na^+^ currents (Fig. 8A, right). In the absence of *Os*CNIH1, the transporter followed the default pathway for exogenous proteins to the oocyte plasma membrane, similarly to what was observed for other heterologous expressed organelle-localized proteins, including aquaporins from plants. This response resembles the unregulated transport of the glutamate receptor (AMPAR) to the plasma membrane observed in the *Caenorhabditis elegans cnih1* mutant (Brockie *et al.*, 2013), and may be a result of an imbalance in the assembly of the COPII system caused by the lack of cornichon which leads to the uncontrolled targeting of *O*sHKT1;3 to the oocyte plasma membrane. Only when the cargo receptor is present is the transporter retained in the endomembrane system (Fig. 8B, centre). Alterations in COPII-mediated ER membrane protein export have also been observed upon overexpression of Sec12p and were proposed to be a result of Sar1p titration (D’Enfert *et al.*, 1989; DaSilva *et al.*, 2004). Yet another result that further supported the interaction between *Os*CNIH1 and *Os*HKT1;3 as being responsible for the proper targeting of the transporter to the Golgi membrane was the restoration of the location of *Os*HKT1;3 in the yeast mutant BY4741*Δerv14* upon co-expression with either of the cornichon homologues, *Os*CNIH1 or Erv14p (Fig. 9A). Parallel to these results, restoration of salt sensitivity to the BY4741*Δerv14* yeast mutant by co-expression of the rice sodium transporter together with either of the two cornichon homologues (Fig. 9B) further supported the dependence of *Os*HKT1;3 on *Os*CNIH1 for the correct targeting of the transporter to its membrane of residence, the Golgi. These results strongly indicated that in the absence of Erv14p, *Os*HKT1;3 was not delivered to the Golgi membrane and, thus, did not disturb the functioning of the cell, allowing yeast growth in the presence of Na^+^.

The presence of *Os*CNIH1 in the ER and the Golgi apparatus (Figs 4, 5) is comparable with that reported for the homologues in yeast (Powers and Barlowe, 1998) and mammals (Harmel *et al.*, 2012). CNIH’s homologues are associated with the transport of membrane proteins, including Axl2p in yeast (Powers and Barlowe, 1998, 2002; Gillingham *et al.*, 2004), Gurken in *Drosophila* (Bökel *et al.*, 2006), and GluRA_o_ in mammals (Schwenk *et al.*, 2009; Kato *et al.*, 2010; Harmel *et al.*, 2012; Herring *et al.*, 2013), and, it is argued, *Os*HKT1;3 in rice. *Os*CNIH1 conserves three out of five amino acids (I96, F97, and L100; Fig. 2B, arrows) in the cytoplasmic loop between TMD2 and TMD3 that effect the binding of Erv14p to COPII vesicles (Powers and Barlowe, 2002), suggesting that in plants, transport of membrane proteins in the early secretory pathway may also involve the association of *Os*CNIH1 with COPII vesicles. This is supported by the co-localization of *Os*CNIH1 with the *bona fide* COPII marker *At*Sec24 in tobacco leaves (Fig. 5B), a site where *Os*CNIH1 would be functioning as a cargo receptor for *Os*HKT1;3 (Fig. 4A). Targeting of *Os*HKT1;3 to the Golgi apparatus by *Os*CNIH1 can be compared with the Erv14p-dependent localization of Rud3p to the Golgi in yeast that also involves the participation of the GTP-binding protein Arf1p (Gillingham *et al.*, 2004). Recent results in yeast have revealed the central role played by Erv14p as a cargo receptor for a number of membrane proteins including the flippases Dnf1p and Dnf2p, the hexose transporters Hxt3p, Hxt4p, and Hxt5p, as well as the Na^+^/H^+^ exchanger Nha1p, among others (Herzig *et al.*, 2012). As indicated by the BiFC results (Fig. 3B), *Os*CNIH1 seems to form oligomers. This feature seems to be shared with cargo receptors such as ERGIC-53 and p24 protein families (Dancourt and Barlowe, 2010) and may be important for its functioning. In particular, Emp47p, a Type I membrane protein with homology to ERGIC-53, has been demonstrated to oligomerize as a requisite for its exit from the ER (Sato and Nakano, 2003). Further studies will help to confirm this result with *Os*CNIH1.

In view of the evidence that cornichons may act as cargo receptors in plants, the Arabidopsis Membrane-based Interactome Network Database (MIND1; Jones *et al.*, 2014) was analysed. It was found that the *Arabidopsis* cornichon *At*CNIH1 (At3g12180) interacts with 535 proteins (Supplementary Table S3 at *JXB* online), with 30% of these corresponding to membrane transport proteins (Supplementary Fig. S5), strengthening the proposed role of CNIH as a membrane protein cargo receptor in plants.

### *Os*HKT1;3 localizes to the Golgi system

Co-localization of *Os*HKT1;3–EYFP with the Golgi membrane marker *Gm*Man1 (Fig. 4C), its apparent aggregation in BFA bodies caused by BFA (Fig. 6C; Ritzenthaler *et al.*, 2002), and the high motility of the punctate structures in which it is observed (Supplementary Fig. S3, Supplementary Video S1 at *JXB* online) are evidence consistent with localization of this transporter at the Golgi membrane. *Os*HKT1;3 does not localize to the plasma membrane (Fig. 6B) as no co-localization with the aquaporin *At*PIP2A was observed, distinguishing this HKT from other members of the gene family (Horie *et al.*, 2007, 2011; Munns *et al.*, 2012). Similar protein fusions of GFP to *At*AMT1;1 (a plasma membrane resident transporter) and to another rice HKT, *Os*HKT1;4, demonstrated that these two proteins localized to the plasma membrane (unpublished results), discarding the possibility that tagging of the protein might have modified its membrane of residence (Figs 4C, 6A, C, D). In plants, transporters belonging to large gene families, as is the case for HKT, have members localized to multiple cellular locations, which can include the Golgi. In particular, NHX5 and NHX6, two Na^+^/H^+^ exchangers (Bassil *et al.*, 2011), as well as a phosphate transporter, PHT4;6 (Cubero *et al.*, 2009), have been localized to the Golgi, whereas other family members are either plasma membrane (i.e. NHX7, NHX8, and PHT1) or tonoplast localized (NHX1–NHX4). Moreover, members of the aquaporin family of proteins have diverse subcellular localizations, being present in several intracellular compartments (Wudick *et al.*, 2009). Therefore, it is likely that HKT transporters may be located in different cellular compartments.

### *Os*HKT1;3 is an Na^+^ transporter

Functional characterization in two different heterologous expression systems; *Xenopus* oocytes and yeast, demonstrated that *Os*HKT1;3 is an Na^+^ transporter (Figs 7–9). Electrophysiological data from the oocyte system showed that the reversal potential changed according to Nernst with extracellular Na^+^ (54.2 mV per decade) (Fig. 7D), indicative of the high selectivity of *Os*HKT1;3 for sodium, confirming results reported previously (Jabnoune *et al.*, 2009). Expression of *Os*HKT1;3 in the yeast BY4741 rendered the colonies more susceptible to Na^+^ (Fig. 9B), probably by generating an ionic imbalance in the cell and further confirming the selectivity of the transporter for this cation.

Although no direct evidence is available, it is proposed that *Os*HKT1;3 in the Golgi membrane could catalyse the downhill transport of Na^+^ towards the cytoplasm and, thus, may function as an alternative shunt conductance for the H^+^ pumps located in this organelle (Mitsuda *et al.*, 2001; Shimaoka *et al.*, 2004; Dettmer *et al.*, 2005; Strompen *et al.*, 2005). The Golgi-located NHX5 or NHX6 Na^+^ exchangers (Yokoi *et al.*, 2002; Bassil *et al.*, 2011) would lead to Na^+^ accumulation within the Golgi, establishing the Na^+^ gradient required for the functioning of *Os*HKT1;3. Recently, an Na^+^-selective mechanism has been demonstrated to play a similar role in the lysosomal membrane of mammalian cells (Wang *et al.*, 2012).

Together, the results demonstrate that the highly selective Na^+^ transporter/channel *Os*HKT1;3 unexpectedly is located at the Golgi membrane, targeted to this endomembrane by its interaction with a newly described cargo receptor in plants, *Os*CNIH1. Finding *Os*HKT1;3 at the Golgi raises the possibility of new functions for the HKT family in addition to those previously described. Future research would demonstrate the biological significance of *Os*CNIH1 and *Os*HKT1;3 in the rice plant.

## Supplementary data

Supplementary data are available at *JXB* online.

Figure S1. Deletion of *ERV14* in *S. cerevisiae.*

Figure S2. Quantification of co-localization between *Os*CNIH1 and *Os*HKT1;3 and other membrane markers.

Figure S3. Dynamics of *Os*HKT1;3–EYFP in tobacco epidermal cells.

Figure S4. Rice HKT transporters do not interact with each other, indicating the absence of oligomerization.

Figure S5. Classification of *At*CNIH-interacting proteins.

Table S1. Primers used for gene cloning into the Gateway (TOPO), pYeP352, and pOO2 plasmids.

Table S2. Primers used for the deletion of *ERV14* in *S. cerevisiae.*

Table S3. *Arabidopsis thaliana* proteins interacting with *At*CNIH1.

Video S1. Dynamics of *Os*HKT1;3–GFP-labelled bodies that resemble the movement associated with the Golgi apparatus.

Supplementary Data
