# Supplementary material for: Identification of rice cornichon as a possible cargo receptor for the Golgi-localized sodium transporter OsHKT1;3
Source: J Exp Bot. 2015 Mar 7;66(9):2733–48. doi: 10.1093/jxb/erv069 (PMC4986874; doi:10.1093/jxb/erv069)
Supplement: Supplementary Data [file supp_66_9_2733__index.html]

Identification of rice cornichon as a possible cargo receptor for the Golgi-localized sodium transporter OsHKT1;3 — Identification of rice cornichon as a possible cargo receptor for the Golgi-localized sodium transporter OsHKT1;3 — Identification of rice cornichon as a possible cargo receptor for the Golgi-localized sodium transporter OsHKT1;3 — Supplementary Data 

# Identification of rice cornichon as a possible cargo receptor for the Golgi-localized sodium transporter OsHKT1;3

## Supplementary Data

Data files

**Files in this Data Supplement:**

- Supplementary Data - Supplementary Data
- Supplementary Data - Supplementary Data
- Supplementary Data - Supplementary Data
