# Supplementary material for: Identification of rice cornichon as a possible cargo receptor for the Golgi-localized sodium transporter OsHKT1;3
Source: J Exp Bot. 2015 Mar 7;66(9):2733–48. doi: 10.1093/jxb/erv069 (PMC4986874; doi:10.1093/jxb/erv069)
Supplement: Supplementary Data [file supp_erv069_jexbot140194_file001.pdf]

Title: Identification of Rice Cornichon as a possible cargo receptor for the sodium transporter *Os*HKT1;3

Authors: Paul Rosas-Santiago[a,b,] Daniel Lagunas-Gómez[a], Bronwyn, J. Barkla[a*], Rosario Vera-Estrella[a], Sylvie Lalonde[b], Alexander Jones[b], Wolf B. Frommer[b], Hana Sychrová[c], Olga Zimmermannova[c], Omar Pantoja[a,1]

SUPPLEMENTARY MATERIAL

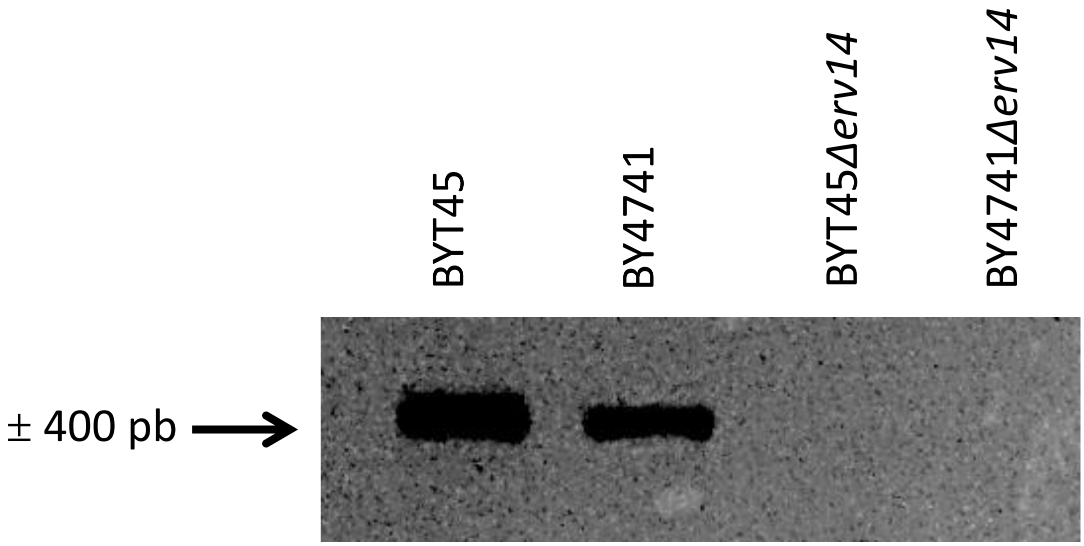

Figure S1. Deletion of *ERV14* in *S. cerevisae*. PCR amplification of the *ERV14* gene from the different yeast cells showing its absence in the BYT454*Δerv14* mutants.

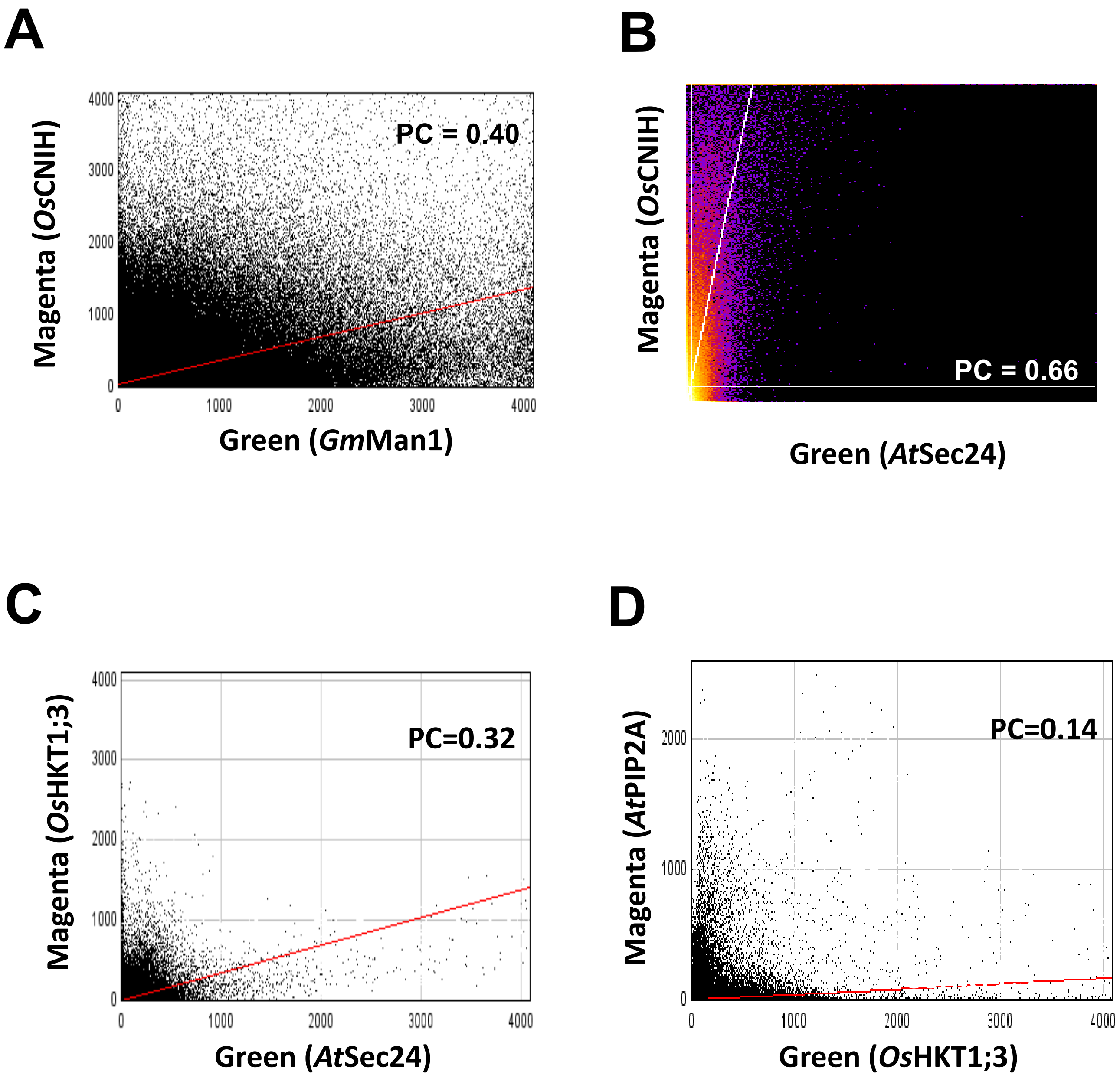

Figure S2. Quantification of co-localization between *Os*CNIH1 or *Os*HKT1;3 with membrane markers. Scatter plots of pixel distribution of the magenta (Y-axis) and green (X-axis) channels employing the Costes algorithm including in the plug-in JACoP (ImageJ Software) for *Os*CNIH1-mCherry and *Gm*Man1-Citrina **A**; *Os*CNIH1-mCherry and *At*Sec24-YFP **B**; *Os*HKT1;3-mCherry and *At*Sec24-YFP **C**; and *At*PIP2A-mCherry and *Os*HKT1;3-EYFP **D**.

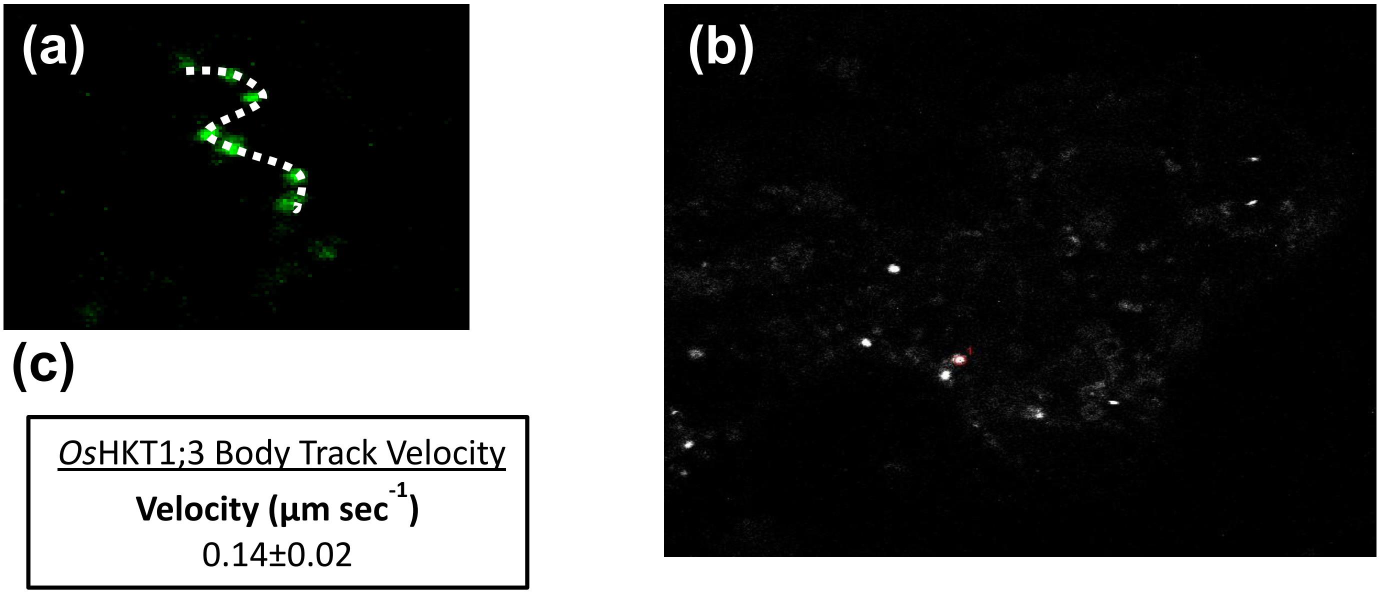

Figure S3. Dynamics of *Os*HKT1;3-EYFP in tobacco epidermal cells. (a) Trajectory of a single *Os*HKT1;3-EYFP body. **(b)** Dynamics of *Os*HKT1;3-EYFP bodies in an epidermal cell. **(c)** Mean velocity of *Os*HKT1;3-EYFP bodies derived from similar data as shown in **(b).** Values correspond to the mean±SD from 9 measurements.

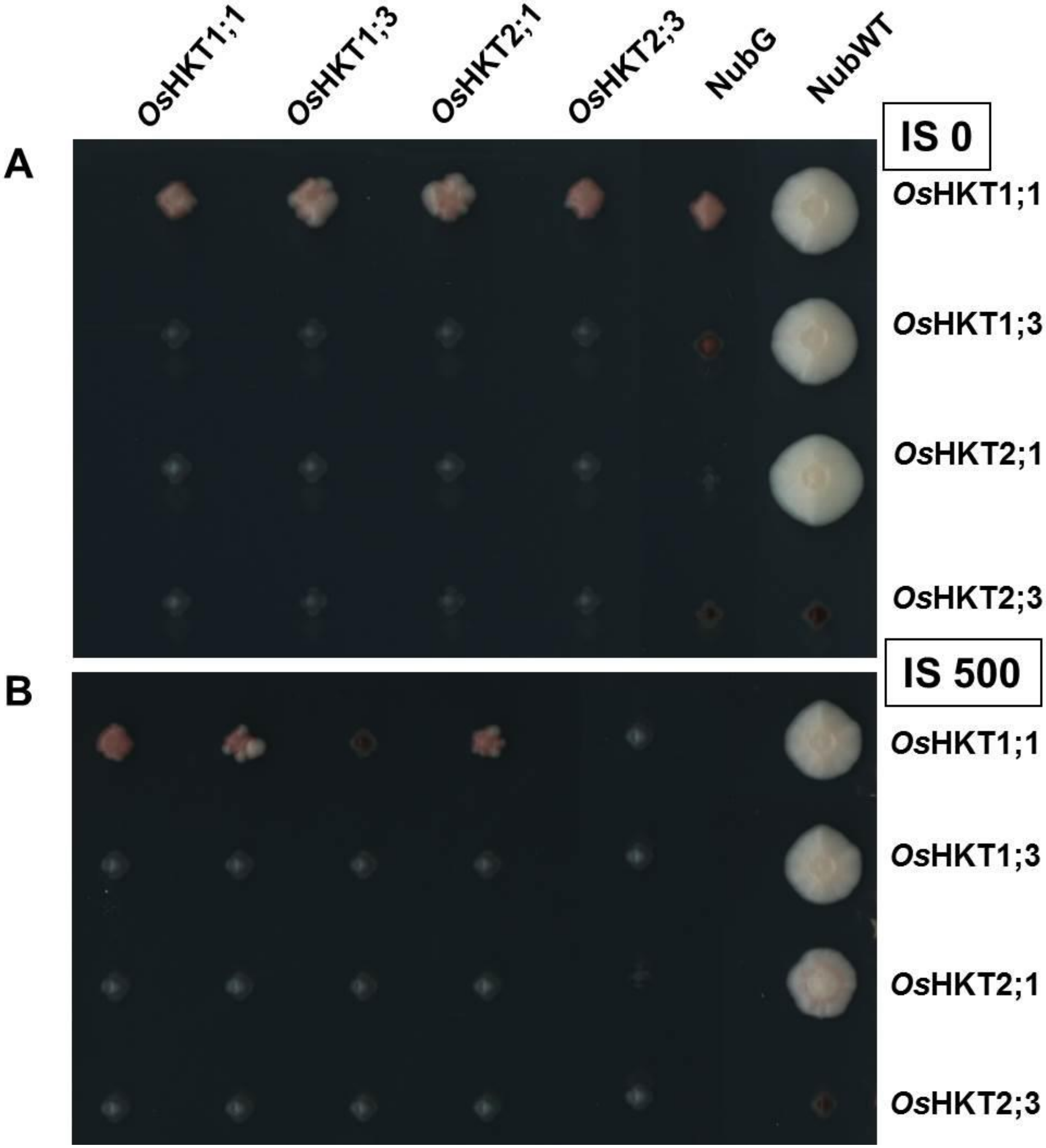

Figure S4. Rice HKT transporters do not interact between them, indicating the absence of oligomerization. OsHKT transporters do not seem to interact between them as indicated by the absence of yeast cell grow in selective medium in the absence (**A**) or in the presence of 0.5 mM methionine (**B**).

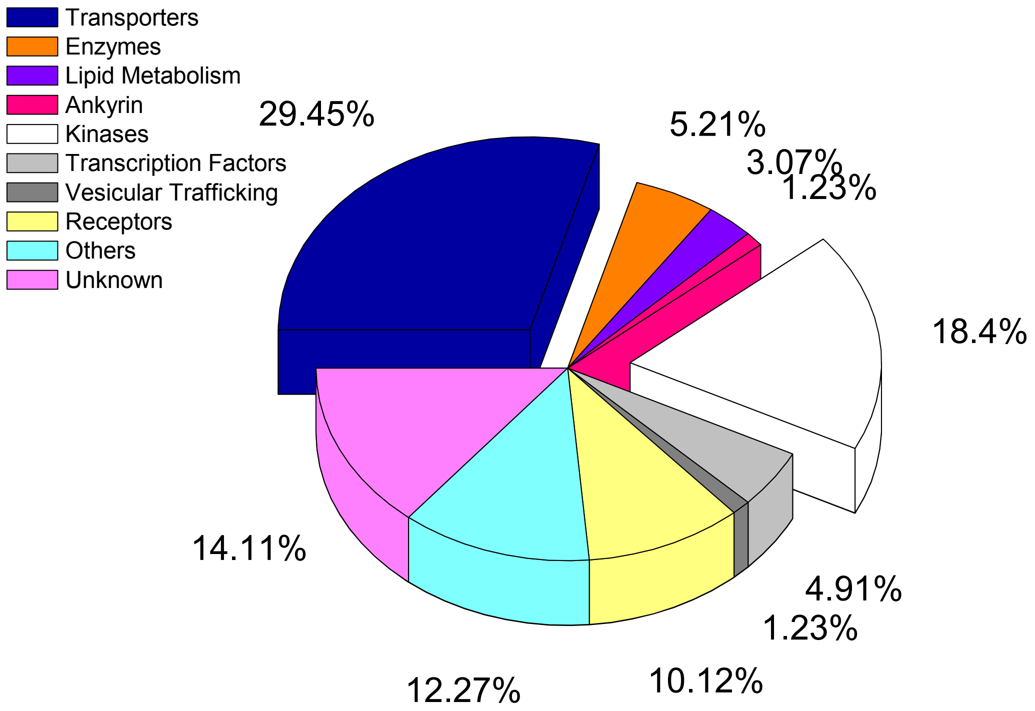

Figure S5. Classification of the proteins interacting with *At*CNIH1. Thirty percent of the proteins included in the Arabidopsis interactome correspond to transporters. Data were obtained from the membrane-base associomics database:
https://associomics.dpb.carnegiescience.edu/Associomics/Home.html
(See also Table S3).

Table S1. Primers Used for gene cloning into the Gateway (TOPO), pYeP352 and pOO2 plasmids.

Gene	Primer Squence (5’→3’)	
	Forward	Reverse
OsHKT1;3	CACCATGAATCATTGTCTTGTAGTA TCCCACAAAAAACTCC	AGAACCACCACCAGAACCACCACCTC TAAGCTTCCAGGCTCTTC
OsCNIH1	CACCATGGTTTTCGTGTGGCT	CTCATCCTCCTCCAATAATACAG
pYeP352-*OsHKT1;3*	GTACATTATAAAAAAAAATCCTGAA CTTAGCTAGATATTATGAATCATTG TCTTGTAG	CACGACGTTGTAAAACGACGGCCAGT GCCAAGCTTGCATGTTATCTAAGCTTC CAGGCTC
pYeP352-*OsHKT1;3-1*	TCC GTA TCT GCA TCA ACT G	
pYeP352-*OsHKT1;3-2*	CTT CAG CAA TCC TAG TCC T	
pOO2-*OsHKT1;3-EGFP*	CGGGATCCATGAATCATTGTCTTGT AGTATCCCACA	CGGAATTCAGAACCACCACCTCTAAG CTTCCAGGCTCTTC
pOO2-EGFP	CGGAATTCGGTGGTGGTTCTATGG TGAGCAAGGGCGAGGA	CCAATGCATTGGTTCTGCAGTTACTTG TACAGCTCGTCCA

Table S2. Primers Used for the deletion of *ERV14* in Yeast.

Gene	Primer Squence (5'→3')	
	Forward	Reverse
ScERV14-kanMX	GTAAAGTAAAAAAATTAAGAATAAAAAGAAAAATGGGTGCTTGGTTCGTACGCTGCAGGTCGAC	CTTTGGATTTCAATGTCTTGTTGGATTAGAAGTCATCACCACAGCATAGGCCACTAGTGGATCTG
ScERV14	CCCATTTCTCACATTTATC	TTTCACAGTCATGCTCACCC
